# Correspondence regarding: Post-traumatic headache: the use of the sport concussion assessment tool (SCAT-3) as a predictor of post-concussion recovery

**DOI:** 10.1186/s10194-017-0795-1

**Published:** 2017-08-31

**Authors:** Salman Khazaei, Shiva Mansouri Hanis, Kamyar Mansori, Olivia Begasse de Dhaem, William B. Barr, Laura J. Balcer, Steven L. Galetta, Mia T. Minen

**Affiliations:** 1 Department of Epidemiology, School of Public Health, Hamadan University of Medical Sciences, Hamadan, Iran; 2 School of Public Health, Shahid Beheshti University of Medical Sciences, Tehran, Iran; 3 Social Determinants of Health Research Center, Kurdistan University of Medical Sciences, Sanandaj, Iran; 4 Department of Epidemiology, School of Public Health, Iran University of Medical Sciences, Tehran, Iran; 5 Department of Internal Medicine, New York University Langone Medical Center, New York, USA; 6 Department of Neurology, Columbia University – New York Presbyterian Hospital, New York, USA; 7 Department of Neurology, New York University Langone Medical Center, New York, USA

## Abstract

This article consists of a Letter to the Editor regarding Post-traumatic headache: the use of the sport concussion assessment tool (SCAT-3) as a predictor of post-concussion recovery, recently published in *The Journal of Headache and Pain*, along with a response from the original authors.

## Letter regarding post-traumatic headache: The use of the sport concussion assessment tool (SCAT-3) as a predictor of post-concussion recovery

Salman Khazaei, Shiva Mansouri Hanis, Kamyar Mansori.

We read the paper entitled “Post-traumatic headache: the use of the sport concussion assessment tool (SCAT-3) as a predictor of post-concussion recovery” written by Begasse de Dhaem et al. which was published in *The Journal of Headache and Pain* December 2017. The aim of the study was to evaluate the use of concussion screening scores in a concussion clinic population to assess for post-traumatic headache. Finally, the results of this study showed the presence and frequency of post-traumatic headache are associated with the SCAT-3 symptom severity score, which is the most important predictor for post-concussion recovery. The SCAT-3 symptom severity score might be a useful tool to help characterize patients’ post-traumatic headache [1]. However, although this research was valuable and the results are interesting, some methodological issues should be considered relating to this cross- sectional study.

Regardless of the results obtained from the model, it should be explained that accurate predictors or determinants of a dependent variable cannot be reliably identified by a cross-sectional study because predictors must be identified based on cohort studies [2, 3]. In other words, predictive or casual inferences cannot be made from cross-sectional studies because of the associations between variables measured at the same time point in such studies. Without the temporality assumption (the dependent variable must occur after the independent variable) there is no way of determining whether a factor is a risk factor, is predictive/causal, or is a consequence of the outcome [4, 5]. Therefore, longitudinal studies are essential for developing assumptions to be used in clinical prediction models, whereas in this study [1], a cross-sectional study was used to identify the independent predictors of post-concussion recover. Therefore, it is essential to interpret the results of this study in light of the above explanation.

## Response from original authors

Olivia Begasse de Dhaem, William B. Barr, Laura J. Balcer, Steven L. Galetta and Mia T. Minen.

Thank you for taking the time to read and think about our article. We agree that predictors cannot be identified from a cross-sectional study. Our study assesses associations between the SCAT 3 and post-traumatic headache. Our cross-sectional study showed that both post-traumatic headache prevalence and frequency are associated with higher SCAT-3 symptom severity and SCAT-3 symptom scores. We hence mentioned that this is an important finding because cohort studies have shown that the SCAT-3 symptom severity score is a predictor of recovery from SRC [6, 7].

## References

[1] De Dhaem OB, Barr WB, Balcer LJ, Galetta SL, Minen MT (2017). Post-traumatic headache: the use of the sport concussion assessment tool (SCAT-3) as a predictor of post-concussion recovery. The Journal of Headache and Pain.

[2] Mansori K, Hanis SM, Shadmani FK (2017) Postpartum modern contraceptive use in northern Ethiopia: prevalence and associated factors-methodological issues in this cross-sectional study. Epidemiology and Health 39:e2017019 10.4178/epih.e2017019 PMC5543293 28774166

[3] Steyerberg E. Clinical prediction models: a practical approach to development, validation, and updating: Springer Science & Business Media, New York. 2008

[4] Hanis SM, Shadmani FK, Mansori K (2017). Letter to editor: the waist circumference-adjusted associations between hyperuricemia and other lifestyle-related diseases: methodological issues in crosssectional study. Diabetology & metabolic syndrome.

[5] Ayubi E, Sani M (2016). Carotid atherosclerosis is associated with left ventricular diastolic function: methodological issue. J Echocardiogr.

[6] Meehan WP, Mannix RC, Stracciolini A (2013). Symptom severity predicts prolonged recovery after sport-related concussion, but age and amnesia do not. J Pediatr.

[7] Meehan WP, Mannix R, Monuteaux MC (2014). Early symptom burden predicts recovery after sport-related concussion. Neurology.

